# Pharmacological interventions in myopia management

**Published:** 2019-05-13

**Authors:** Tim Fricke, Helena Hurairah, Yuqin Huang, Suit May Ho

**Affiliations:** 1 Senior Research Fellow and Paediatric Optometrist: Brien Holden Vision Institute, Sydney, Australia.; 2 Consultant Ophthalmologist (Paediatrics and Strabismus): Brunei Eye Centre, RIPAS Hospital, Bandar Seri Begawan, Brunei, Darussalam.; 3 Lecturer and Optometrist: Ngee Ann Polytechnic, Singapore.; 4 Education Program Manager and Optometrist: Brien Holden Vision Institute, Melbourne, Australia.

**Daily low-dose atropine has been shown to result in a reduction of >50% in myopia progression over 3 years – with little to no rebound effect.**

**Figure:**
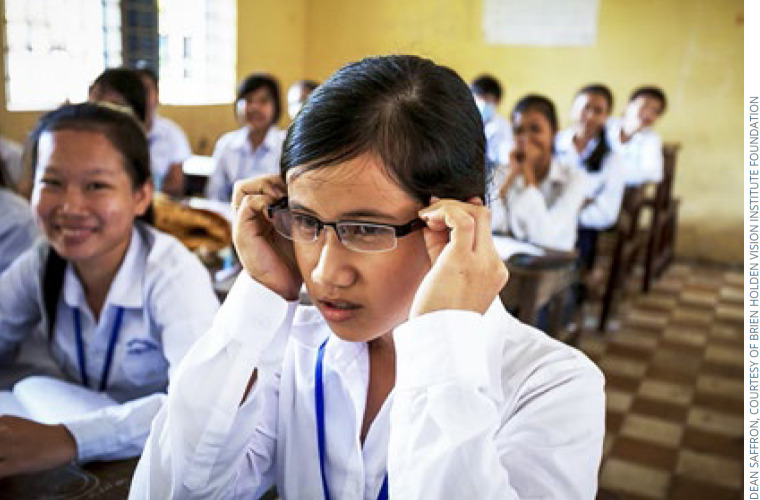
Myopia can get worse very quickly. Drugs such as low-dose atropine can help. CAMBODIA

The long-term burden of myopia on the health care system, the global economy and individual quality of life requires that we do what we can to avoid or delay myopia onset and, when myopia is present, that we provide optical correction and do our best to slow its progression. Behaviour modifications such as increased time outdoors and reduced near work appear useful in avoiding or at least delaying onset of myopia, but have not been shown to slow progression once myopia is present. There is evidence that optical interventions (pp. 19–20) and the pharmacological options presented here can reduce myopia progression in children. This may reduce the risk of vision impairment later in life from complications such as retinal detachment and myopic macular degeneration.

## Daily low-concentration atropine

Atropine is known for its cycloplegic and pupil dilation effects. It cannot correct the blurred vision caused by myopia, but appears to have myopia control effects that are probably mediated in the retina or sclera. This means that doses too small to cause side effects or other symptoms can still reduce myopia progression.

High quality (large cohort, randomised, masked and controlled) trials of 0.01%, 0.025% and 0.05% atropine eye drops have demonstrated significant myopia control effects over 1 year of daily use.^1^,^2^

Comparison of the ATOM studies suggests that 0.01% atropine reduces myopia progression (as measured by spherical equivalent refraction) by >50% over 3 years (2 years of use, then 1 of non-use).^1^ At present, it is less clear to what extent 0.01% atropine reduces myopia progression in terms of axial length. This is an important distinction, because we suspect that axial length is a stronger determinant than spherical equivalent of the lifelong risk of complications such as retinal detachment and myopic macular degeneration. However, further studies are required to confirm this.

With 0.01% atropine, there was little to no rebound effect (i.e., increased myopia progression) during the year of non-use.^1^ The potential rebound effects of other low concentrations (0.025% and 0.05%) are not known.^1^,^2^

Depending on uveal pigmentation and individual sensitivity, all three dosages (0.01%, 0.025% and 0.05%) have subclinical effects on pupil size and accommo-dation, and are therefore very well tolerated.^1^,^2^

## Low-concentration atropine: unresolved questions

Unsettlingly, we do not know exactly how atropine reduces myopia progression. Atropine has a dose-related effect on accommodation, pupil size, dopamine levels in the retina, and scleral fibroblast activity. Any or all of these mechanisms potentially explain atropine's myopia progression effect, as this effect also appears to be atropine dose-related, as does post-treatment rebound. However, retinal dopamine levels and/or scleral fibroblast activity appear the most likely candidates.

## Low-concentration atropine: the challenges

Low-concentration atropine needs to be compounded in most countries, making it too expensive for many people who would benefit from it. It is also commonly an ‘off-label’ use – meaning reduction in myopia progression was not the reason atropine was approved for use in most countries, which can cause insurance and payment issues. Absence of a local compounding pharmacy can be overcome, as compounding pharmacists commonly post or courier drops to patients, even between countries, depending on drug importation rules.

The biggest problem remains cost. Pharmaceutical companies have not shown an interest in manufacturing low-concentration atropine commercially, because of regulatory hurdles in the countries they are most likely to make profits in. This may change if preferred dosage and axial length efficacy questions are resolved.

We have not observed any significant complications from daily low concentrations of atropine, and none have been reported. However, given the potential complications of atropine generally, caution is warranted. Practitioners should note contra-indications (e.g., Down's Syndrome or spastic paralysis), provide patient information (including critical information about complications), advise sun protection if prescribing concentrations above 0.02%, and advise to return immediately or attend a hospital emergency department if signs of adverse reactions are observed.

## Recommendation

Given the current lack of evidence about the potential rebound effect of other concentrations of low-dose atropine, the only treatment regimen we can safely recommend is **0.01% atropine, one drop in each eye, once a day** (preferably at night, just before bed). We recommend reviewing patients one week after the initiation of atropine use to assess visual function (including distance visual acuity, refraction, accommodation accuracy, and pupil reactions) and to check for any adverse reactions.

After that, it is sensible to regularly review a child with progressive myopia as usual, e.g., every 6–12 months. The practitioner should have clear criteria for ceasing atropine management (e.g., a wash-out period after each 2 years of use, as used in the ATOM studies).^2^ Various groups are actively investigating the effects of a range of low-concentration atropine regimens on myopia progression. Recommendations may change as future studies are published.

## Warning: avoid regular-concentration atropine

We *do not* recommend the use of daily regular-concentration atropine. Although 1% atropine is widely available, and high-quality randomised controlled trials of 0.1%, 0.5% and 1% atropine eye drops have demonstrated strongly significant myopia control effects over 2 years of daily use,^1^,^3^ there is a significant rebound effect. One year after ceasing 2 years of daily 1% atropine, study participants showed almost as much cumulative myopia progression as if atropine had never been used.^1^ Several options for reducing the rebound effect have been proposed (e.g. tapering atropine in either concentration or frequency of use), but none has been shown safe in prospective randomised controlled trials.

**Other candidates**

**Pirenzepine.** The use of pirenzepine 2% twice a day has been shown to have a significant myopia control effect over 2 years^4^ There have been no studies about progression once treatment has stopped, so there is no information about a potential rebound effect. We are unaware of any jurisdictions in which pirenzepine is commercially available.

**7-methylxanthine.** 7-methylxanthine (7-mx) appears to exhibit a safe, significant myopia control effect in both animals and humans.^5^ As is the case with pirenzepine, there have been no studies about progression once treatment has stopped, so there is no information about a potential rebound effect. Denmark is the only jurisdiction we are aware of in which 7-mx is available and permitted for use.

## Conclusion

Daily, low-concentration atropine appears safe and beneficial, with 0.01% atropine leading to a reduction in myopia progression of >50% over 3 years, as measured by spherical equivalent refraction. This has the potential to reduce the frequency of high myopia by over 73%^6^ and to reduce the risk of visual impairment associated with high myopia.

Even so, questions remain about the mechanism of action, efficacy (e.g., does it moderate the progression of axial elongation?), the optimal dosage for myopia control, the effect of place and/or ethnicity on optimal dosage, and how atropine interacts with other myopia control interventions (e.g., does combining atropine with dual focus contact lenses or bifocal spectacles result in additive myopia control, does it multiply the effect, or do they interfere with each other?).

We advocate the evidence-based role of daily low-concentration atropine in moderating myopia progression, so long as drug cessation guidelines are employed and there is a clear administrative framework and patient safety protocols.

In our opinion, this currently means considering a prescription of 0.01% atropine for use in both eyes, once per day, at night, in appropriate patients.
